# Junior medical students’ knowledge about and attitudes towards electroconvulsive therapy in a South African setting

**DOI:** 10.4102/sajpsychiatry.v23i0.1062

**Published:** 2017-07-03

**Authors:** Matthew B. Mausling, Muiruri Macharia, Gerhard P. Jordaan

**Affiliations:** 1 Department of Psychiatry, Faculty of Medicine and Health Sciences, Stellenbosch University, South Africa

## Abstract

**Background:**

Although electroconvulsive therapy (ECT) is a safe and effective treatment modality with a long history of use in psychiatry, it remains controversial owing to misconceptions and negative attitudes among the public and medical profession. The aim of this study was to explore the state of knowledge and attitudes towards ECT among a sample of South African medical students.

**Method:**

Prior to their theoretical psychiatry module, 131 second-year medical students responded to an anonymous online survey designed to assess the source and extent of their ECT knowledge as well as their attitude towards ECT and psychiatry in general.

**Results:**

The Internet (46.6%) and TV and/or movies (30.5%) were the principal sources of knowledge of ECT while ‘professional publication’ was the least common (0%). The students’ attitudes towards psychiatry were generally positive and nearly one-third (29.8%) would consider specialising in the field. Overall, perception towards ECT was mixed, with many respondents approving of its use albeit only as a last resort. Notably, low ECT knowledge scores were associated with more negative attitudes towards this treatment modality and a lower perception of psychiatry as a medical speciality.

**Conclusion:**

The findings indicate that for these students, media is the main source of ECT knowledge. While they are generally knowledgeable about ECT, they still harbour some misconceptions and negative attitudes about the treatment. Knowledge appears able to amend these attitudes, thus underlining the importance of integrating accurate information about ECT into the preclinical medical curriculum rather than leaving it to mass media to forge warped perceptions and attitudes for these future clinicians.

## Introduction

Electroconvulsive therapy (ECT) is a technique used to treat mental illnesses by applying electric current through the brain to induce generalised seizures. It is used worldwide as one of the most effective treatments for several treatment-resistant psychiatric disorders, particularly major depressive disorder, which forms about 80% of patients treated with this procedure.^1^ ECT is also used, albeit less frequently, to treat schizophrenia, catatonia and acute mania.^2^ It is used as a first-line therapy in life-saving emergencies, when there is a history of good response, when perceived risk is less than using medication such as in pregnancy, or when the patient opts for ECT over medication.^2,3^

Convulsive therapy was first introduced in 1934 by Meduna who used chemically induced seizures to treat schizophrenia.^4^ The modern form using electricity was developed by Ugo Cerletti and Lucio Bini in 1938 and was administered without anaesthesia or muscle relaxants (unmodified).^5^ This form was associated with serious complications including fractures, dislocations and dental injury and was gradually replaced by the safer ‘modified’ ECT from the early 1950s.^6,7^ The use of ECT subsequently flourished throughout the world, becoming the mainstay of biological treatments for psychiatric disorders from the 1940s to the 1960s.^6^

The use of ECT gradually declined from the 1970s, such that in the USA, for example, only 36 558 patients were treated in 1986, compared to 58 667 in 1975.^8,9^ The decline was largely because of two main reasons: first was the introduction of pharmacotherapy for severe mental disorders, which partly relegated ECT from first-line to last-resort treatment for medication-resistant and severe conditions.^8^ Second was the negative portrayal (largely based on the ‘unmodified’ form) of the treatment as an inhumane procedure, raising public concern and anti-ECT lobbying and legislation.^1^ Two psychiatrists, Thomas Szasz and his former student Peter Breginn, were among the most outspoken anti-ECT activists. Szasz did not consider mental illnesses to be true diseases and was of the view that it would therefore be inappropriate to treat with medical interventions such as ECT.^10^ Breggin’s criticism rests on his view that psychiatric medications and ECT have deleterious effects that outweigh any benefit and should be replaced with psychotherapy, empathy and love.^11^ Although not based on empirically sound arguments, anti-ECT activism has persisted and ECT remains one of the most stigmatised, controversial and misunderstood procedures among the public and medical practitioners.^1,12,13^

The ambivalence and misinformation about ECT among medical personnel is largely a consequence of inadequate education in medical schools.^14,15^ It is therefore important that medical students should be well informed about ECT as they represent a major future force in shaping public opinion and influencing the level of support the treatment might expect in the professional space.

A key reason for assessing the views and attitudes of medical students towards ECT is to inform teaching of medical curricula by uncovering deficits and areas that can be addressed to reduce the stigma against ECT and promote its perception by the public and non-psychiatric health professionals.

Evidence from several countries indicates that medical students generally have negative attitudes towards ECT which, however, can be improved through knowledge. These attitudes are better when the source of knowledge is a health professional,^12^ thus highlighting the significance of how students obtain their information. In this study, we report the results of a survey on the attitude of South African medical students without prior exposure to a formal didactic experience on ECT. Our aim was to assess the student’s attitudes towards ECT and how the attitude relates to the source and level of knowledge on the treatment. In cognisance of the decline in the popularity of psychiatry as a discipline, we also assessed how the attitudes towards psychiatry relate to ECT knowledge among these students.

## Methods

All second-year medical (MB, ChB) students (*n* = 259) were invited via email to participate in the anonymous online survey in the months of October and November, 2015. The MB, ChB is a 6-year programme involving theoretical rotations from the first year and clinical rotations from the third year. The invitation email contained a consent and participation information sheet outlining the aims and objectives of the study along with a link to the online questionnaire. The survey was based on a self-selected sample as only those who accepted our request participated in the survey. Anonymity was assured as there were no identifiers used. The online questionnaire was live for 8 weeks and a repeat email was sent every 2 weeks. Once completed, students could not resubmit a new or alternative questionnaire.

The survey was designed and released using SUrvey, an online-based survey tool developed by Stellenbosch University and maintained by the university’s Department of Information Technology. The content and format of the questions were based on previous similar studies. In addition to sex and age, the participants were asked to identify their main source of information on ECT in the first section. The next five questions assessed their general attitude towards psychiatry, which was rated on a Likert Scale as was the next 10-question section assessing their attitudes towards ECT. The last section had 10 ‘true/false’ questions evaluating their knowledge of ECT.

Frequency counts and percentages were used to summarise qualitative variables, while chi-square test was used to compare them within gender categories. Analysis of the variance was used to compare mean knowledge scores across the three response categories for the ECT and/or psychiatry attitudinal questions.

## Ethical considerations

Ethics approval was obtained from the University of Stellenbosch Human Research Ethics Committee to conduct the survey at the Tygerberg Medical campus.

## Results

### General characteristics and main sources of knowledge of electroconvulsive therapy

The survey was completed by 131 (50.5%) of the 259 second-year medical students invited to participate. The mean (standard deviation) age of the respondents was 20.4 (1.8) years and the majority (69.0%) were women. The main sources of knowledge of ECT are shown in Table 1. Overall, the Internet (46.6%) and TV and/or movies (30.5%) were the principal sources. Other sources of knowledge of ECT that were identified included lectures (12.2%), explanation by a professional (4.6%) and experience of ECT (4.6%) while ‘professional publication’ was the least common, with no respondent identifying with it.

**TABLE 1:** Main source of knowledge of electroconvulsive therapy.

Variables	Overall *n* (%)	Male *n* (%)	Female *n* (%)
Internet	61 (46.6)	21 (51.2)	40 (44.4)
TV and/or movies	40 (30.5)	10 (24.4)	30 (33.3)
Lecture	16 (12.2)	6 (14.6)	10 (11.1)
Explanation by another doctor	6 (4.6)	3 (7.3)	3 (3.3)
Experience of friend and/or family	6 (4.6)	1 (2.4)	5 (5.6)
Explanation by psychiatrist	2 (1.5)	0 (0)	2 (2.2)
Professional publication	0	0	0

### Attitudes towards psychiatry

Attitude towards psychiatry was generally positive with no significant gender differences (Table 2). The majority of respondents agreed that psychiatry is an important medical discipline (88.5%) warranting inclusion in the MB, ChB curriculum (88.5%). Although the majority thought that it has a scientific basis (62.6%) and that psychiatric medication is effective (61.1%), only a third of female respondents (33.3%) and about one-fifth of male respondents (22.0%) would consider specialising in the discipline.

**TABLE 2:** General attitudes towards psychiatry.

Variables	Overall	Male	Female	*p*
**Psychiatry is an important discipline**				
Disagree	3 (2.3)	0(0)	3 (3.3)	
Agree	116 (88.5)	34 (82.9)	82 (91.1)	0.063
Neutral	12 (9.2)	7 (17.1)	5 (5.6)	
**Include Psych in MB, ChB curriculum**				
Disagree	4 (3.1)	2 (4.9)	2 (2.2)	
Agree	116 (88.5)	34 (82.9)	82 (91.1)	0.36
Neutral	11 (8.4)	5 (12.2)	6 (6.7)	
**Would consider specialising in psychiatry**				
Disagree	54 (41.2)	19 (46.3)	35 (38.9)	
Agree	39 (29.8)	9 (22)	30 (33.3)	0.43
Neutral	38 (29)	13 (31.7)	25 (27.8)	
**Psychiatry has scientific basis**				
Disagree	9 (6.9)	4 (9.8)	5 (5.6)	
Agree	82 (62.6)	23 (56.1)	59 (65.6)	0.50
Neutral	40 (30.5)	14 (34.1)	26 (28.9)	
**Psych medication is effective**				
Disagree	11 (8.4)	5 (12.2)	6 (6.7)	
Agree	80 (61.1)	20 (48.8)	60 (66.7)	0.14
Neutral	40 (30.5)	16 (39)	24 (26.7)	

Results are presented as *n* (%).

### Attitudes towards electroconvulsive therapy

Responses to questions regarding attitudes towards ECT were mixed and gender was not a significant factor (Table 3). The majority of respondents disagreed that ECT should be illegal to perform (51.9%) but thought it should only be used as a last resort (48.9%). The largest proportion did not think that ECT is cruel (39.7%) or a form of punishment (71.8%), or that it can cause permanent brain damage (37.4%) or death (38.2%). Many of the respondents, however, would not have ECT if it was recommended to them (42.0%), would not recommend it to their patients (44.3%) and would not be comfortable performing it even after receiving adequate training (40.5%).

**TABLE 3:** Students’ attitudes towards electroconvulsive therapy.

Questions	Overall	Male	Female	*p*
**Would have ECT if recommended to me**				
Disagree	55 (42.0)	15 (36.6)	40 (44.4)	
Agree	35 (26.7)	8 (19.5)	27 (30.0)	0.10
Neutral	41 (31.3)	18 (43.9)	23 (25.6)	
**Would recommend ECT to my patients**				
Disagree	58 (44.3)	18 (43.9)	40 (44.4)	
Agree	23 (17.5)	4 (9.8)	19 (21.1)	0.21
Neutral	50 (38.2)	19 (46.3)	31 (34.4)	
**Would feel comfortable administering ECT if adequately trained**				
Disagree	53 (40.5)	13 (31.7)	40 (44.4)	
Agree	45 (34.3)	14 (34.1)	31 (34.4)	0.22
Neutral	33 (25.2)	14 (34.1)	19 (21.1)	
**ECT is cruel**				
Disagree	52 (39.7)	13 (31.7)	39 (43.3)	
Agree	22 (16.8)	7 (17.1)	15 (16.7)	0.41
Neutral	57 (43.5)	21 (51.2)	36 (40.0)	
**ECT causes pain**				
Disagree	35 (26.7)	7 (17.1)	28 (31.1)	
Agree	41 (31.3)	14 (34.1)	27 (30.0)	0.24
Neutral	55 (42.0)	20 (48.8)	35 (38.9)	
**ECT is used to punish aggressive or uncooperative patients**				
Disagree	94 (71.8)	26 (63.4)	68 (75.6)	
Agree	11 (8.4)	5 (12.2)	6 (6.7)	0.33
Neutral	26 (19.8)	10 (24.4)	16 (17.8)	
**ECT is unsafe and may result in death**				
Disagree	50 (38.2)	14 (34.1)	36 (40.0)	
Agree	18 (13.7)	4 (9.8)	14 (15.6)	0.42
Neutral	63 (48.1)	23 (56.1)	40 (44.4)	
**ECT causes permanent brain damage**				
Disagree	49 (37.4)	15 (36.6)	34 (37.8)	
Agree	21 (16.0)	6 (14.6)	15 (16.7)	0.93
Neutral	61 (46.6)	20 (48.8)	41 (45.6)	
**ECT should only be used as a last resort**				
Disagree	26 (19.8)	6 (14.6)	20 (22.2)	
Agree	64 (48.9)	20 (48.8)	44 (48.9)	0.51
Neutral	41 (41.3)	15 (36.6)	26 (28.9)	
**ECT should be illegal to perform**				
Disagree	68 (51.9)	19 (46.3)	49 (54.4)	
Agree	13 (9.9)	4 (9.8)	9 (10.0)	0.65
Neutral	50 (38.2)	18 (43.9)	32 (35.6)	

Results are presented as *n* (%).

ECT, electroconvulsive therapy.

### Knowledge of electroconvulsive therapy

Responses to questions regarding knowledge on ECT are presented in Table 4. The overall mean knowledge score was 74.7% (14.9) and there was no significant difference between the genders. The questions with lower scores were mostly related to the technical aspects of administering ECT. For example, only 39.7% of respondents knew that the usual course of ECT was more than four sessions and only 57.3% knew that an anaesthetic is administered prior to administration of ECT. However, an overwhelming majority of the respondents knew that ECT involves passing an electrical current through the brain (99.2%), not usually administered daily (95.4%) and is used to treat conditions other than depression (94.7%).

**TABLE 4:** Students’ knowledge regarding electroconvulsive therapy.†

Variables *n* (%)	Overall	Male	Female	*p*
ECT was first used in the 1930s	81 (61.6)	29 (70.7)	52 (57.8)	0.18
ECT involves passing an electrical current through the brain	130 (99.2)	41 (100)	89 (98.0)	0.99
ECT is not only used to treat depression	124 (94.7)	38 (92.7)	86 (95.6)	0.68
Anaesthetic is given during ECT	75 (57.3)	23 (56.1)	52 (57.8)	0.99
A muscle relaxant is given prior to the administration of ECT	89 (67.9)	28 (68.3)	61 (67.8)	0.99
The usual course of ECT is more than four sessions	52 (39.7)	13 (31.7)	39 (43.3)	0.25
ECT should not be administered daily	125 (95.4)	41 (100)	84 (93.3)	0.18
Successful ECT-induced seizure does not need to be for ≥ 5 min	100 (76.9)	30 (73.2)	70 (78.7)	0.51
ECT does not result in permanent memory loss	101 (77.7)	32 (78.0)	69 (77.5)	0.99
ECT does not cause personality changes	65 (50.4)	18 (43.9)	47 (53.4)	0.35
Knowledge score (mean, s.d.)	74.7 (14.9)	74.9 (14.7)	74.6 (15.1)	0.91

Results are presented as *n* (%) except where specified as mean (s.d.).

ECT, electroconvulsive therapy; s.d., standard deviation.

† , The above scores represent proportion of respondents scoring correctly on each question.

### Relationship between electroconvulsive therapy knowledge score and attitude towards electroconvulsive therapy

The relationship between participants’ ECT knowledge scores and attitudes towards the treatment is shown in Table 5. Low mean ECT knowledge score was generally associated with less favourable attitudes towards ECT. Compared to respondents in disagreement, significantly lower scores were noted in students agreeing that ECT is cruel (78.8% vs. 65.9%; *p* = 0.002), causes pain (78.3 vs. 69.0; *p* = 0.011) and could result in permanent brain damage (79.0 vs. 65.7; *p* = 0.002) or death (79.4 vs. 63.3; *p* < 0.0001). Mean ECT scores were also significantly different in responses to the questions of ECT being used as a punishment for uncooperative patients and as a last-resort treatment.

**TABLE 5:** Students’ electroconvulsive therapy knowledge scores across electroconvulsive therapy attitudinal questions.†

Questions	Disagree	Agree	p*
Would have ECT if recommended to me	72.4	77.4	0.273
Would recommend ECT to my patients	73.1	77.4	0.483
Would feel comfortable administering ECT if well trained	71.3	76.9	0.107
ECT is cruel	78.8	65.9	0.002
ECT causes pain	78.3	69.0	0.011
ECT is used as a punishment for uncooperative patients	77.3	56.3	< 0.0001
ECT is unsafe and may result in death	79.4	63.3	< 0.0001
ECT causes permanent brain damage	79.0	65.7	0.002
ECT should only be used as a last resort	75.4	74.2	0.940
ECT should be illegal to perform	77.8	60.8	0.001

ECT, electroconvulsive therapy.

† , The above scores represent the percentage obtained in the knowledge score questions.

* , The *p* values are for post hoc analyses of the variance between ‘agree’ and ‘disagree’. ‘Neutral’ column is excluded.

### Relationship between electroconvulsive therapy knowledge score and attitude and psychiatry

Students who viewed psychiatry as an ‘important’ discipline had significantly higher (*p* = 0.004) mean ECT knowledge scores compared to those who did not (Table 6). ECT knowledge scores were not significantly different on the three questions pertaining to scientific basis of psychiatry, its inclusion in the medical curriculum or effectiveness of medication. However, those opting to remain neutral to the question of considering specialising in psychiatry had a significantly lower mean ECT knowledge score (69.7; *p* = 0.048) compared to those disagreeing (77.2%) or agreeing (75.9%).

**TABLE 6:** Students’ mean electroconvulsive therapy knowledge scores across psychiatry attitudinal questions.

Questions	Neutral	Disagree	Agree	*p*
Psychiatry is an important discipline	81.7	50.0	74.6	0.004
Include psychiatry in MB, ChB curriculum	72.7	67.5	75.1	0.551
Would consider specialising in psychiatry	69.7	77.2	75.9	0.048
Psychiatry has scientific basis	73.3	71.1	75.7	0.527
Psychiatry medication is effective	76.0	73.6	74.1	0.790

## Discussion

This survey assessed the knowledge and attitudes towards psychiatry and ECT among medical students without formal psychiatry training at a South African medical school. The students’ attitudes towards psychiatry were generally positive and their indicated preference for it as a potential career choice was quite high. Most students obtained their information about ECT from mass media – specifically the Internet and TV, with fewer students obtaining their knowledge of ECT from lectures and professionals. The least common source of knowledge was from professional publications. Overall, perception towards ECT was mixed, with many respondents approving of its use but only as a last resort. Finally, and most notable, low ECT knowledge scores were overwhelmingly associated with more negative attitudes towards this treatment modality.

Attitude towards psychiatry was generally positive and, in contrast to numerous past findings as reviewed by Lyons,^16^ the positivity was matched with enthusiasm as a career choice. In nearly all the studies in that review, the proportion of students in favour of a career in psychiatry was generally below 10%. Thus, the proportion in our sample (30%) was comparatively high, especially when viewed with the awareness that general practitioners, surgeons, paediatricians, gynaecologists, physicians, pathologists and all the other medical specialisations will come from this group. The rate in our study is consistent with observed fluctuations in interest of a psychiatry career throughout the 6 years of medical school. The proportion is often high during the first and second years, declines after the first clinical rotation, and then rises after the psychiatry internship before gradually decreasing until the completion of medical school.^17,18^

As in previous studies, mass media remained the most popular source of information,^19,20,21^ but we specifically identified the Internet, rather than TV, as the single most important source. This is not surprising, given the increasing prominence of technology in knowledge acquisition but is of concern because of the media’s largely negative depictions of ECT contrasting the often-positive views about ECT among treatment recipients and their families.^22,23,24^ Because the media has tremendous influence over public consciousness, repeated presentation of negative stereotypes perpetuates misconceptions and contributes to the stigma of ECT and mental illness. A review of 20 films that refer to or depict ECT found that earlier films depicted ECT as a severe but helpful remedy for psychological distress but that the depiction has changed over the last few decades to that of a brutal, harmful procedure with little or no therapeutic benefit. The authors concluded that negative attitudes about ECT among patients, families, health professionals and the general public stem in large part from films.^25^ Indeed, Ken Kesey’s 1962 book (and its film adaptation in 1975), *One Flew Over the Cuckoo’s Nest* is credited with irredeemably denting the public perception of ECT and hastening its demise as a treatment of choice for mental illness.

It is therefore worthwhile encouraging medical students to utilise scientific sources of information. Yet none of the students cited journals/professional literature as their main source of knowledge, which is nonetheless unsurprising as these learners were yet to receive formal lectures in psychiatry. The limited use of academic journals by undergraduate medical students, despite an awareness of these sources, has been previously documented.^26^ It could be a cue to incorporate reading tasks in medical curricula that stimulate the use of journal articles. The students’ overall knowledge of ECT was good, especially regarding its basic aspects, and nearly all respondents were aware of the technique’s working principle and utility. There were, however, areas of concern. About 32% and 43% of the future doctors did not know that a muscle relaxant and a general anaesthetic, respectively, are used during ECT. In addition, half of the sample incorrectly associated ECT with personality changes.

The students’ attitude towards ECT was more mixed and less confident with ‘neutral’ making 30% – 48% of the responses in all but two section questions. Most students committing to a polarised response approved the use of ECT but many suggested it should only be a last resort. This is possibly an extension of a widely entrenched but inaccurate presumption that ECT should only be used in medication-resistant patients.^27^ The misconception was also reported in high proportions in previous surveys.^14,28,29^ Although most students in our study generally considered ECT to be safe and unlikely to result in death or long-term ill effects, more than half (54%) of those not giving a neutral answer still viewed it as a painful procedure. Perhaps this explains why many of them would not consider undergoing, recommending or performing an ECT with confidence even when adequately trained. These attitudes appear to be deep seated and were not amended by a positive attitude towards psychiatry. For example, of the 116 respondents viewing psychiatry as an ‘important’ discipline, only 22 (19%) would suggest it to their patients while almost half (46%) of those who would consider specialising in psychiatry still believed ECT should only be used as a last resort. However, it was noteworthy that low knowledge of ECT was overwhelmingly associated with less favourable attitudes towards ECT, which supports previous reports correlating positive attitudes towards ECT with clinical experience and knowledge of the procedure.^30^ It is also possible that this relationship between attitudes and knowledge of ECT may be bidirectional, that is, while negative attitudes of ECT may account for the poor knowledge of ECT, preconceived negative attitudes towards ECT could also subvert interest to improve knowledge of ECT. Nonetheless, the findings indicate that attitudes may be improved through education, suggesting that programmes aimed at dispelling myths and combating stigma in and towards psychiatry could be beneficial. That higher ECT knowledge was associated with perception of psychiatry as an essential speciality may also point to a role of education in boosting interest in psychiatry as a career.

### Limitations

The present study had some limitations. It only involved students from one medical school and therefore the findings cannot be extrapolated to all medical students in the country. Furthermore, the response rate was quite modest (50.5%), thus casting a shadow on the representativeness of the sample. The final sample size, however, compared well with most other similar studies.

## Conclusion

The results provide insights on how junior South African medical students, without formal exposure to psychiatry training, perceive psychiatry as a career and ECT as a treatment modality. The students view psychiatry positively, both as a discipline and as a potential career. While they were generally knowledgeable about ECT, they have reservations and still harbour misconceptions about the treatment. It appears that knowledge/education can produce some changes to the students’ attitudes regarding ECT and, by extension, psychiatry, which underline the importance of integrating accurate information about ECT into the preclinical medical curriculum rather than leaving it to mass media as the main source of knowledge and perception for these future clinicians. Given that these students had no formal exposure to psychiatry, it would be useful to establish how psychiatry education would influence their knowledge and attitude towards ECT and psychiatry in general.
